# Origin, evolution, and tissue-specific functions of the porcine repetitive element 1

**DOI:** 10.1186/s12711-022-00745-3

**Published:** 2022-07-27

**Authors:** Min Zheng, Tianfu Guo, Bin Yang, Zhiyan Zhang, Lusheng Huang

**Affiliations:** State Key Laboratory for Pig Genetic Improvement and Production Technology, Jiangxi Agricultural University, Nanchang, China

## Abstract

**Background:**

The porcine repetitive element 1 (PRE1) is the most abundant short interspersed nuclear element (SINE) in the *Sus scrofa* genome and it has been suggested that some PRE1 can have regulatory functions. The million copies of PRE1 in the porcine genome have accumulated abundant CpG dinucleotides and unique structural variations, such as direct repeats and patterns of sequence degeneration. The aims of this study were to analyse these structural variations to trace the origin and evolutionary pattern of PRE1 and to investigate potential methylation-related functions of PRE1 based on methylation patterns of PRE1 CpG dinucleotides in different tissues.

**Results:**

We investigated the evolutionary trajectory of PRE1 and found that PRE1 originated from the ancestral CHRS-S1 family through three main successive partial duplications. We found that the partial duplications and deletions of PRE1 were likely due to RNA splicing events during retrotransposition. Functionally, correlation analysis showed that the methylation levels of 103 and 261 proximal PRE1 were, respectively, negatively and positively correlated with the expression levels of neighboring genes (Spearman correlation, *P* < 0.01). Further epigenomic analysis revealed that, in the testis, demethylation of proximal PRE1 in the *HORMAD1* and *HACD3* genes had tissue-specific enhancer and promoter functions, while in the muscle, methylation of proximal PRE1 repeats in the *TCEA3* gene had an enhancer function.

**Conclusions:**

The characteristic sequences of PRE1 reflect unique patterns of origin and evolution and provide a structural basis for diverse regulatory functions.

**Supplementary Information:**

The online version contains supplementary material available at 10.1186/s12711-022-00745-3.

## Background

Transposable elements (TEs) constitute more than 40% of the *Sus scrofa* genome [1–3]. They have been increasingly considered to be important genomic elements for organizing genome architecture, regulating gene expression, and affecting genome size, diversity, and evolution. Comparative genomics analysis has shown that TEs are extensively co-opted for the regulation of host genes [4]. Human and animal model studies have clarified that TEs act as *cis*-regulatory elements to regulate gene expression [4–9]. They often play cell- and species/clade-specific *cis*-regulatory roles in the modulation of gene expression through diverse mechanisms [10].

Short interspersed nuclear elements (SINEs) are retrotransposons with a high GC content and thus represent DNA methylation hotspots that can have major effects on the transcription of neighboring genes [11]. Tissue culture models have provided mounting evidence for the target-specific action of 5-methylcytosine (5mC) [12]. The methylation of SINEs or Alu elements is a highly dynamic process during human development and ageing. Methylation and demethylation of proximal Alu elements can regulate the expression of neighboring genes. For instance, hypermethylated Alu elements in the promoter of the *MIEN1* gene leads to the inhibition of its expression [13].

In recent years, researchers have attempted to identify and characterize SINEs in pigs. Porcine repetitive element 1 (PRE1) accounts for 88.5% of the TEs in the pig genome, with more than one million copies [14]. PRE1 belongs to the tRNA-derived *suiform*-specific SINEs [15–17] and diversification of its sequence occurred at least 43.2 million years ago [18]. A typical PRE1 element consists of three distinct parts: the head, the body, and the tail [17]. The head contains bipartite elements of the RNA polymerase III promoter (A-box and B-box), the body harbours two short direct repeats, and the tail consists of a poly(A) tail of variable length [15]. It has been suggested that PRE1 originated from a type of SINEs called CHRS in the Suina lineage (CHRS-S) (the CHRS family of SINEs derived from tRNA^Glu^ in a common ancestor of the lineages of *Cetaceans*, *Hippopotamuses*, *Ruminants* and *Suiformes*) [19] and then expanded through three successive events during the first half of the Tertiary period [16], including insertion of a tRNA^Arg^-related sequence, insertion of a 19-bp sequence, and duplication of a 28-bp sequence [20]. The expanded PRE1 elements are grouped into 15 types or lineages (Pre0_SS, PRE1_SS, PRE1a, b, c, d, d2, e, f, f2, g, h, i, j, and k) [14], most of which are inactive, with only PRE1a (3%) showing a weak activity [16].

Although the structure of the different PRE1 elements is clear, little is known about their evolution and biological functions. In this study, our aims were to: (1) reconstruct the evolutionary history of the PRE1 elements; (2) clarify their functions by investigating the methylation pattern of proximal PRE1 in the muscle and the testis using high-depth whole-genome bisulfite sequencing (WGBS); and (3) to attempt to identify *cis*-regulatory functions of PRE1 by combining RNA-seq and ChIP-seq data for differentially-methylated regions.

## Methods

### Animals and samples

*Longissimus dorsi* and testis tissues from eight adult male Bamaxiang pigs were used in this study. Feeding conditions and sampling methods were as described in Zheng et al. [21]. All procedures involving animals were in compliance with the guidelines for the care and use of experimental animals established by the Ministry of Agriculture of China, and the trial was approved by the Animal Ethics Committee at Jiangxi Agricultural University (No. JXAULL-2016001).

### Whole-genome bisulfite sequencing and RNA-seq

Sixteen samples from eight individuals were subjected to WGBS and RNA-seq following the standard procedures. After quality control (reads quality > Q20), the WGBS data were aligned to the *Sus scrofa* 11.1 genome assembly using the bwa-meth-master algorithm with default parameters [22], and the Picard (MarkDuplicates) and SAMTools (rmdup) software were used for removal of PCR duplicates [23]. MethylDackel0.3.0 (https://github.com/dpryan79/MethylDackel) was used to summarize the methylation status of the sequences with mapping scores ≥ 20. The RNA-seq data were mapped to the *Sus scrofa* 11.1 genome assembly with the HISAT2.1.0 program [24], then the StringTie software [24] was used to assemble the reads into merged transcripts, and the FeatureCounts software to quantify gene expression levels [25]. The expression levels of each transcript were quantified as fragments per kb of transcript per million mapped reads (FPKM). The raw WGBS and RNA-seq reads are deposited in the China National Center for Bioinformation database (PRJCA003221, https://bigd.big.ac.cn/). The ongoing Functional Annotation of Animal Genomes (FAANG) Project shared the unpublished regional Bam files from ChIP-seq [26], miRNA-seq and Iso-seq. Mapping depth was extracted using SAMtools [27] and visualized with the Integrative Genomics Viewer (IGV) tool [28].

### Calibration and PRE1 annotation

PRE1 lineage consensus sequences were extracted from the SINEBase database (http://sines.eimb.ru/) [17]. To determine the length of the head–body consensus sequence for PRE1, 1000 copies of PRE1 were identified from *Sus scrofa* 11.1 by using RepeatMasker (http://genome.ucsc.edu/) and compared. The consensus sequence (231 nt) was then mapped to *Sus scrofa* 11.1 by BLASTn [29] to detect all the copies of PRE1 in the porcine genome.

The following step-wise approach was used to calibrate the exact coordinates of the PRE1 and to search their target site duplications (TSD). First, we extracted a ~ 600-nt sequence, including a ~ 200-nt aligned PRE1 element and a 200-nt sequence on each side. Second, we took a continuous k-mer of 8 nt from PRE1 to map the start position to the 5′ end, and mapped the k-mers to the 200-nt 3′ end sequence until an exact match. Third, the mapped k-mer was extended until a mismatch was identified, and the longest mapped k-mer was considered as a TSD. The distance between the two TSD was considered to be the length of the PRE1. Fourth, we used the AAAA sequence to distinguish the head–body part from the tail part of the PRE1. If no TSD was identified in the target sequence, we used the conservative GGAGTTCCC and poly(A) sequences to determine the length of the head–body part. Finally, we annotated the calibrated PRE1 based on *Sus_scrofa.Sscrofa11.1.98.gtf*. Distances from the midpoints of the intergenic PRE1 to their neighbouring genes were also calculated. Proximal PRE1 and distal PRE1 were defined as those that were 1 to 2 kb and 2 to 100 kb upstream of their flanking genes, respectively.

### Sequence analysis

We used the MEGA7 software [30] to perform multiple sequence alignment. Obvious alignment errors were corrected manually. Neighbour-joining trees were built using MEGA7. The nucleotide diversity and base change per site between two sequences (D) were calculated using DnaSP6 [31]. The secondary structures of PRE1 RNA were predicted using the mfold web server (http://unafold.rna.albany.edu/?q=mfold) [32]. We estimated differentiation times following Nei’s function ($t=D/2\alpha$), with *α* = 4.6 × 10^–9^ [33].

The *Needleman–Wunsch* genetic distances between homologous PRE1 sequences were calculated using a custom Python script. The genetic distance matrix among sequences was used as the input data for a principal component analysis (PCA), using the PCA function from the FactoMineR R-package [34]. Results were visualized using the ggplot2 R-package [35].

The sequences of human tRNA and SINEs were obtained from Vassetzky and Kramerov [17]. Six *Suidae* genome resequencing datasets were downloaded from public databases under accession numbers ERP001813 [16] and SRA096093 [36], and the reads was mapped to the *Sus scrofa* 11.1 genome assembly, using bwa [22]. We used SAMtools [27] to extract the mapping depth and IGV as the genome browser.

### Enrichment analysis of splicing signals on PRE1

First, we annotated all the calibrated PRE1 based on known exon information and selected the PRE1 that partially overlapped with the exons. Second, PRE1 overlapping with 3′ UTR, 5′ UTR, or RNA gene sequences were filtered out, and the sequences of PRE1 that overlapped with coding DNA sequences (CDS) of the reference genome were extracted. Third, we marked the splicing sites on PRE1. Finally, we summarized the distribution of splice sites on the consensus sequence of PRE1 by multiple sequence alignment and manual correction. We obtained 188 splicing signals in the 92-bp direct repeat region of PRE1. Hot splicing sites were defined as having at least six splicing signals at one site on the consensus sequence. The threshold was calculated by 10,000 random samplings using the sample function from the base R-package by setting x = 1:93, size = 188, and replace = True. This resulted in a probability of 0.01 of having more than six splicing signals at one site, which was used as the significance threshold.

### Differential analysis

CpG with a read depth less than 10 and more than 300 and CpG with individual missing rates higher than 50% were filtered out. Then, we calculated the average methylation level for each CpG and gene within the filtered dataset. The Wilcoxon signed rank test was used for the differential analysis between muscle and testis tissues, using a significance threshold of 0.01. We further filtered the differentially methylated PRE1 based on previously reported differentially-methylated regions (DMR) between muscle and testis tissues [21].

## Results

### Characterization of PRE1

**Fig. 1:**
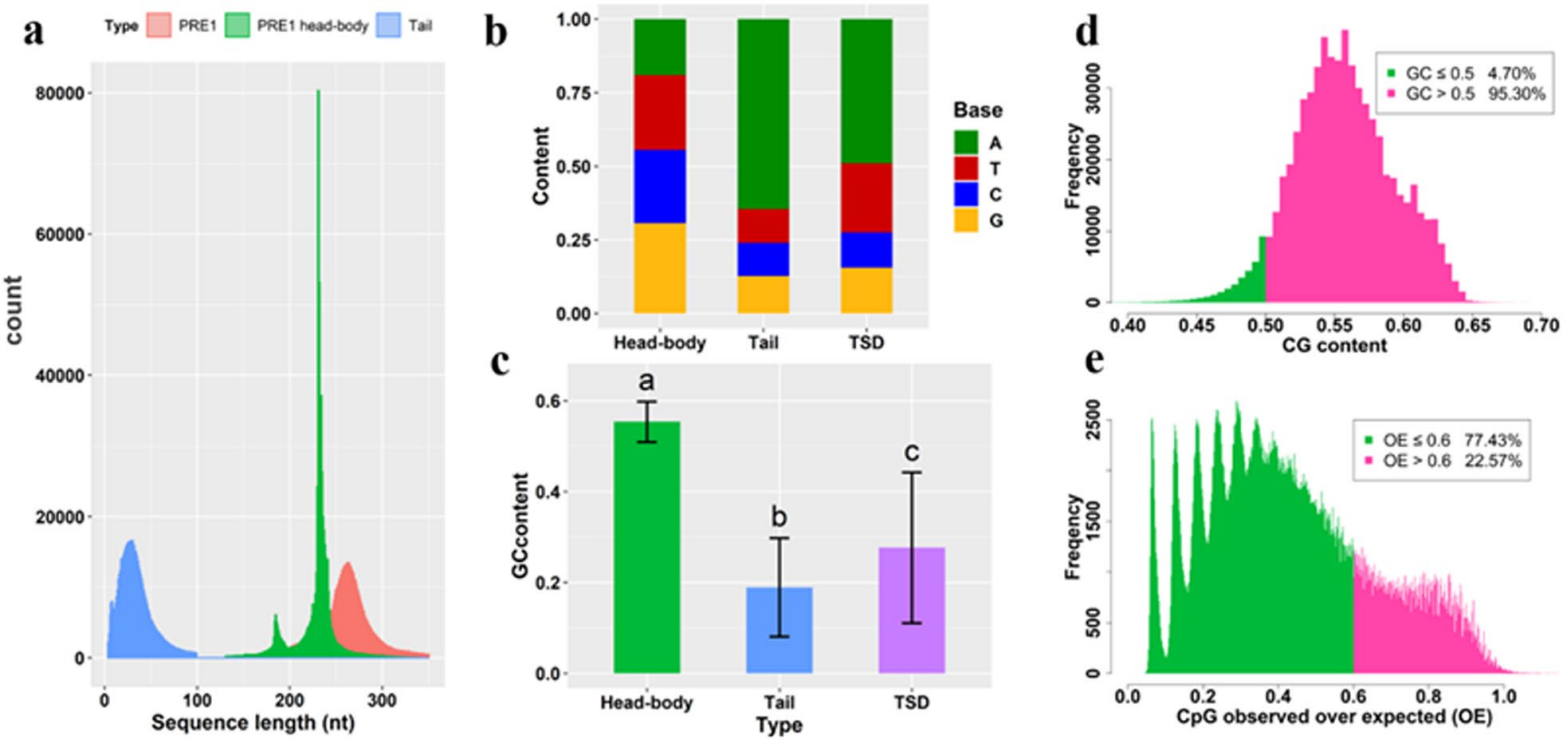
Characterization of PRE1. **a** Length distribution of whole PRE1, head–body, and tail. **b** Nucleotide content of the head–body, tail, and target site duplications (TSD). **c** GC content of the head–body, tail, and target site duplications (TSD). Different letters above the bars indicate a significant difference (*P* < 0.01). **d**, **e** Distribution of the CG content and OE values in the head–body

Although PRE1 have been fully annotated and imported into three main databases (NCBI, Ensembl, and UCSC), their physical locations and local sequence features are not accurately annotated. Here, we recalibrated 626,582 PRE1 and their TSD. Our results show that the average full length of PRE1 was ~ 264 bp, with the distribution of the head–body lengths centered mainly at 231 bp (class I) and 185 bp (class II), and the variable length of the A-rich tail ranging from 10 to 50 bp (Fig. 1a). We also detected tandem PRE1 and nested PRE1 (a PRE1 inserted into another PRE1) (see Additional file 1: Fig. S1a–d), and we observed that target site duplications were present at both ends of tandem PRE1, which suggests that part of the nested or tandem PRE1 may undergo transposition into a new genomic position (see Additional file 1: Fig. S1e). The results indicate that PRE1 have accumulated many indels and structural variations.

The three regions of PRE1 (head–body, tail, and TSD) have different nucleotide contents (Fig. 1b). The GC content in the head–body region of PRE1 (0.56) was approximately twice that in the tail (0.19) and in the TSD (0.28) (Fig. 1c, d, CV = 0.07). In addition, 22% of the head–body sequences exhibited observed/expected CpG (OE) values above 0.6 (Fig. 1e). These results suggest that approximately 235,000 PRE1 may act as potential CpG islands on the basis of the following criteria: a minimum length of 200 bp, a minimum GC content of 50%, and an OE value above 0.6 [37]. In addition, we found that two known swine microRNA sequences are PRE1-derived miRNA, i.e. ssc-miR-4331-1 (chr6:65468483-65468562 bp) and ssc-mirR-4331-2 (chr14:132597139-132597207 bp).

### Origin and structure of PRE1

**Fig. 2:**
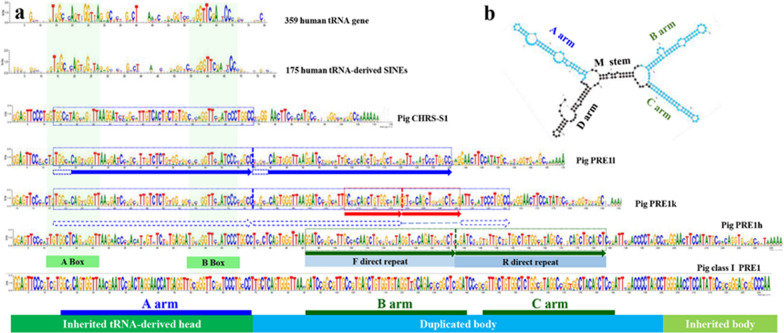
Structure of PRE1. **a** Origin, evolution, and structure of PRE1. The green shadows show the highly conserved A box and B box within the bipartite RNA polymerase III promoter elements. The colored arrows show the observed direct repeats in different PRE1 lineages. The colored boxes on the sequences show the proposed duplication sequences. **b** X-type secondary structure of PRE1 RNA. The sequences of the A, B, and C arms are marked in panel (**a**). The colored B and C arms show the secondary structures of the F direct repeat and R direct repeat, respectively

A previous study reported that PRE1 originated from the CHRS SINE and that during the evolution of PRE1, a region homologous to tRNA^Arg^ was inserted into its head region [20]. However, our analysis found that sequences in the head region are aligned with the head regions (RNA polymerase III promoter) of pig CHRS-1, human tRNA-derived SINE, and human tRNA genes (Fig. 2a, green shadow), which implies that the head region of PRE1 is inherited from CHRS-S1. Thus, the previous assumption that a tRNA^Arg^-related sequence is inserted into the CHRS head is probably incorrect [20].

To clarify the origin of PRE1, we reconstructed its evolution by comparing the consensus sequences between different porcine PRE1 lineages and pig, bovine and caprine CHRS, which showed the presence of repetitive conserved motifs in PRE1k. Then, we carefully compared the conserved motifs to the original CHRS-S1 sequence to uncover their origin. Importantly, this analysis suggested that two partial duplication events occurred to generate PRE1k from CHRS-S1 (Fig. 2a, blue dotted arrows and red arrows in PRE1k) and that a transitional subtype must have occurred between CHRS-S1 and PRE1k. Based on this hypothesis and sequence length data, we successfully identified this transitional subtype in the reference genome and named it PRE1l (Fig. 2a). The observed sequence signatures suggest that these two partial duplication events occurred during the evolution from CHRS-S1 to PRE1k, i.e. a duplication of an approximately 60-bp sequence of CHRS-S1 resulting in PRE1l (Fig. 2a, blue arrows in PRE1l), followed by a duplication of an approximately 18-bp sequence of the PRE1l leading to the ancestral PRE1k (Fig. 2a, red arrows in PRE1k).

Currently, class I PRE1 elements are thought to be quasi-dimeric SINEs that include two internal duplications [17]. Our results showed that there are two tandem 46-bp front (F) and rear (R) direct repeats in the body region (Fig. 2a), which updated previous knowledge of the existence of a 13-bp gap between two 34 bp direct repeats [15]. The third 46-bp duplication in the ancestral PRE1h (Fig. 2a, green arrows, F and R direct repeats) may accelerate the rate of transposition and self-evolution [14, 16]. In addition, we identified dozens of quasi-oligomeric PRE1, including three 46-bp tandem direct repeats (see Additional file 1: Fig. S2a). We also observed other internal duplications in different PRE1 (see Additional file 1: Fig. S2b). These results show that the occurrence of internal duplications has been an important mechanism for the evolution of PRE1. Further functional analysis of the duplication events showed that they seemed to be related to the X-type secondary structure of PRE1 RNA (Fig. 2b).

**Fig. 3:**
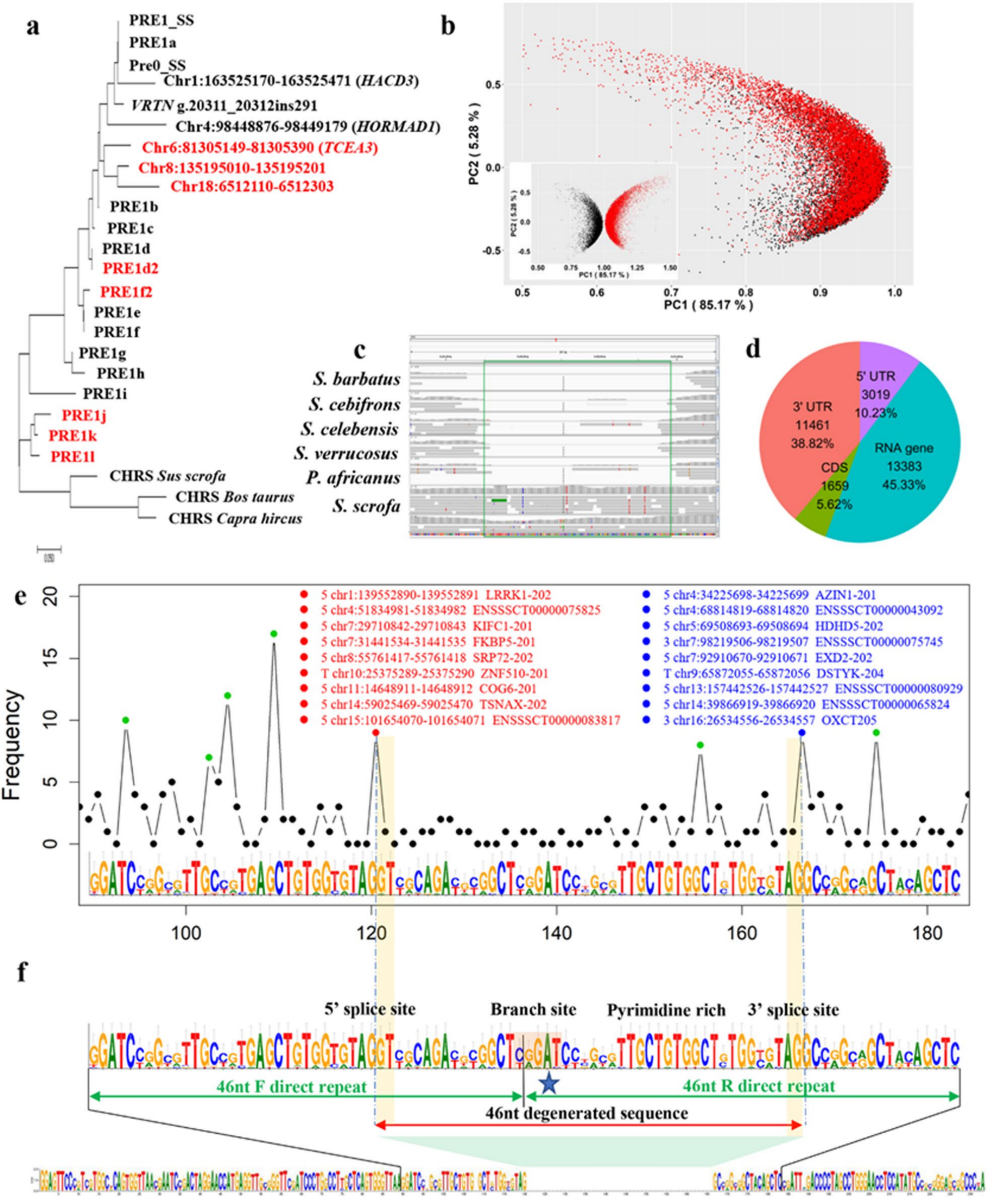
The splicing model of PRE1. **a** Neighbour-joining tree based on consensus sequences of 15 PRE1 lineages, several affected PRE1, and consensus sequences of CRHS from *Sus scrofa*, *Bos taurus* and *Capra hircus*. The PRE1 shown in black font belong to class I, and those in red belong to class II. **b** Principal component analysis based on the Needleman–Wunsch genetic distance from PRE1 to the consensus sequences of 15 PRE1 lineages. Red and black dot sets represent class I and class II PRE1, respectively. The small image at the bottom left shows the specular mapping (x = 1) of class I PRE1. **c** IGV window of the promoter region of the *TECA3* gene. The green box shows the PRE1 region in *Sus scrofa*. The six resequencing reads data downloaded from public databases, *Sus barbatus* (ERR173177) [16], *Sus cebifrons* (ERR173209) [16], *Sus celebensis* (ERR173210) [16], *Sus verrucosus* (ERR173211) [16], *Phacochoerus africanus* (ERR173203) [16], *Sus scrofa* (11-146,206) [36]. **d** Number of PRE1 that overlap with known exons. **e** Splicing signals are enriched in direct repeats. The x axis represents the base position on the consensus sequence. The font colour indicates the type of splicing signal, the physical locations of the splicing signals, and the transcript names. Numbers 3 and 5 represent the 3′ splicing signal and 5′ splicing signal in PRE1, respectively, and T represents the 3′ splicing signal in the reverse complementary sequence of PRE1. **f** Splicing model for the PRE1 body and degenerate sequences

In summary, PRE1 originated independently from CHRS-S1 by three successive partial duplications without insertion of exogenous sequences. Molecular clock model analysis indicated that the first two duplication events occurred at least 51.6 million years ago (Mya) and the third duplication event at least 42.9 Mya, i.e. between the differentiation of *Cetartiodactyla* and the emergence of *Suiformes* [16, 19]. Based on the evolutionary tree of PRE1 (Fig. 3a) and the common structural features (head, body, and tail) with SINEs, we conclude that PRE1 are composed of an RNA polymerase III promoter head (A box and B box), a body that is inherited from the CHR family and that includes a tRNA-related duplicated part, and a poly(A) tail (Fig. 2a, the bottom colour band).

### Splicing models for the evolution of PRE1

Another intriguing question related to the structure of PRE1 is the origin of the approximately 46-bp degenerated direct repeat in PRE1d2 and PRE1f2 (http://sines.eimb.ru/ and (see Additional file 1: Fig. S2c) [17]; the length of the lost sequences is equal to the difference between the lengths of class I and class II PRE1 (Fig. 1a). We also detected a PRE1 element in the promoter of the *TECA3* gene [21] that had also lost a 46-bp sequence from PRE1b [chr6:81305149-81305390 bp, Fig. 3a and (see Additional file 1: Fig. S2c)]. To further investigate the presence of degenerate 46-bp sequences in the PRE1 body, we extracted all subtypes of PRE1 from class II (185 bp long without direct repeats) and constructed their evolutionary tree (Fig. 3a). The Neighbour-joining tree showed that parts of class II PRE1 and PRE1a or PRE1b are orthologous. Furthermore, we randomly extracted 10,000 PRE1 from class I and class II and calculated the *Needleman–Wunsch* genetic distances to the consensus sequences of 15 PRE1 lineages. Principal component analysis (PCA) showed that the evolutionary trajectories of these two PRE1 classes were consistent with each other (Fig. 3b), which further confirmed the orthologous relationship of class I and class II PRE1. Comparative genomic analysis revealed that the transposon activity of class II PRE1 was maintained throughout the differentiation of *Suidae* (11–2.5 Mya, Fig. 3c). Taken together, these results illustrate that the evolution of class I subtype PRE1 was accompanied by the degeneration of a 46-bp sequence in the body region.

This observation implies that a recurrent mechanism underlies the loss of a similar segment (approximately 46 bp) in the body region of class I subtype PRE1. Given that PRE1 is a retrotransposon, we analysed the sequence characteristics of PRE1 by RNA annotation. As expected, we found that a large number of PRE1 elements partially overlapped with known exons, which indicates that PRE1 sequences carry splicing sites (Fig. 3d). Furthermore, we found that the splicing sites were enriched in the F and R direct repeat regions (Fig. 3e, “Methods” section). Each direct repeat carries an AG|GU splice site (Fig. 3e), which is noteworthy, as it can be used as both a 5′ end and a 3′ end splice site. This implies that a splicing event can potentially occur between the two dual-function splicing sites in the posttranscriptional PRE1 body. Furthermore, a branch site was found in the junction region of two direct repeats (Fig. 3f), which is similar to CTGAT in humans and mice [38–40]. Therefore, we hypothesize that the missing 46 bp between the two dual-function splicing sites were eliminated during posttranscriptional processing following the GU-AG rule (Fig. 3f), after which the processed PRE1 were reinserted into the genome by retrotransposition.

**Fig. 4:**
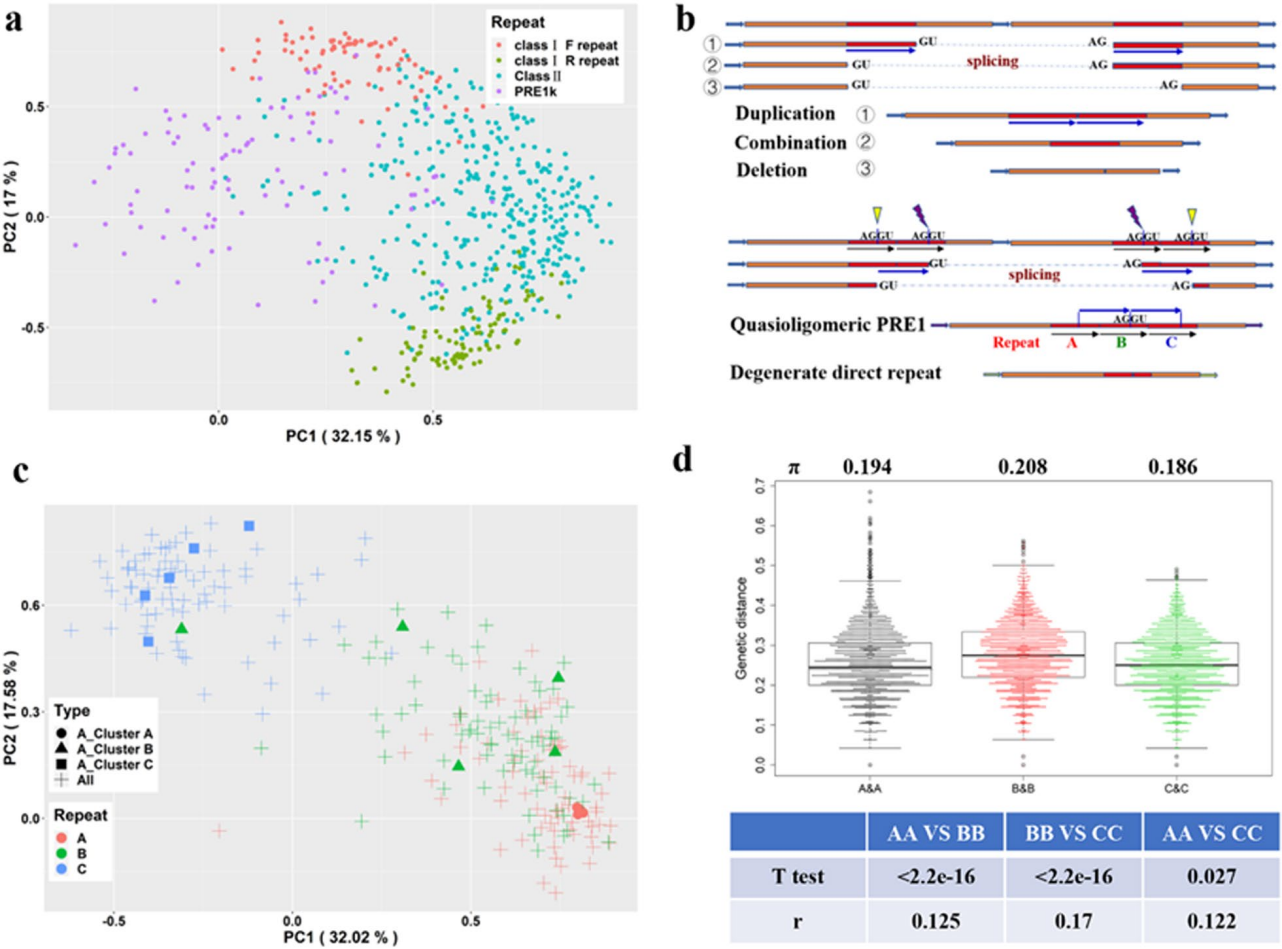
Duplication and deletion mechanisms in the splicing model. **a** PCA based on the genetic distances among different direct repeats from the class I PRE1, class II PRE1, and PRE1k lineage. We extracted the sequences in PRE1k and class II PRE1 that were homologous to the two direct repeats in class I PRE1, and we used the genetic distance matrix among these homologous sequences for PCA. The PRE1k represented the homologous cluster before the direct repeats were formed, and the class II PRE1 represented the homologous cluster after removal of a 46-bp sequence from the ancestral class I PRE1 by splicing. **b** Splicing model of tandem PRE1 for partial duplications and deletions. The top panel shows the three basic models for the formation of three different combined PRE1 types. The bottom panel shows that two tandem quasi-dimeric PRE1 form a combined quasi-oligomeric PRE1 or a combined PRE1 with degenerate direct repeats. **c** Genetic distance of the three direct repeats (repeat A, repeat B, and repeat C) from 82 quasi-oligomeric PRE1 based on principal component analysis (PCA). The genetic distance matrix among the three direct repeats was used for PCA. Repeat B cluster was between the repeat A and C clusters that supported the splicing model for the formation of quasi-oligomeric PRE1. Solid geometric symbols highlight five quasi-oligomeric PRE1 that are the most similar A repeats. **d** Genetic distances between repeat A, repeat B, and repeat C sequences from different quasi-oligomeric PRE1. π, nucleotide diversity; r, Pearson’s correlation coefficient. T test, the *P* values of paired T tests of genetic distances among quasi-oligomeric PRE1 in three direct repeats

According to this splicing model, the preserved repeat sequences actually represent a chimaera of F and R direct repeats of the parental PRE1, rather than the degeneration of a single direct repeat (Fig. 3f); multiple sequence alignment showed that the splicing model had the highest alignment score [e.g. PRE1d2, (see Additional file 1: Fig. S3)]. Since the missing fragment is similar to the single direct repeat, the observed sequence of the derived class II PRE1 seems to be a degenerate whole direct repeat from class I PRE1. To further verify this hypothesis, we randomly selected 500 sequences that are homologous to F or R direct repeats from class II PRE1 and selected 100 PRE1 from class I and PRE1k as control sequences. PCA results based on *Needleman–Wunsch* genetic distances between these homologous sequences were consistent with the splicing model (Fig. 4a).

The splicing model can also explain the partial duplications of PRE1, which can arise by splicing of the post-transcriptional tandem PRE1 to form a new combinatorial PRE1 element (Fig. 4b). Similarly, it is also possible to generate new PRE1 subtypes by alternative splicing (Fig. 4b). To further validate the hypothesis of the formation of partial duplications by splicing events, we analysed the evolutionary relation of the three tandem direct repeats (repeats A, B, and C) of 82 quasi-oligomeric PRE1 (see Additional file 1: Fig. S2a). The PCA results showed that repeat C was independent from repeats A and B and that the genetic distances between repeats A and B were shorter than those between repeats B and C (Fig. 4c). To analyze the genetic relationships between the three repeats, we identified five PRE1 which shared very similar repeat A sequences but had fairly distinct B and C repeats (Fig. 4c). The results showed that the quasi-oligomeric PRE1 did not originate from the same ancestor and that the three duplication sequences did not originate from a single PRE1. The nucleotide diversity (π) of the repeat B cluster was higher than that of the other two clusters, and the relationships within the cluster of B repeats were significantly stronger than those within the clusters of A or C repeats (paired T test, *P* < 2.2 × 10^–16^, Fig. 4d). We speculate that the B repeats are combinatorial repeats and further propose a splicing-based mechanism for the possible formation of quasi-oligomeric PRE1 (Fig. 4b, bottom).

### Methylation level of proximal PRE1

The multiple partial duplications of tRNA-related promoter sequences and the abundant CG dinucleotides imply that PRE1 include many regulatory elements. Moreover, at least one copy of PRE1 was located 2 kb upstream for one-third of the genes and PRE1 have accumulated many variations (1000 randomly selected copies, nucleotide diversity π = 0.136). Thus, PRE1 provide potential *cis*-regulatory elements.

**Fig. 5:**
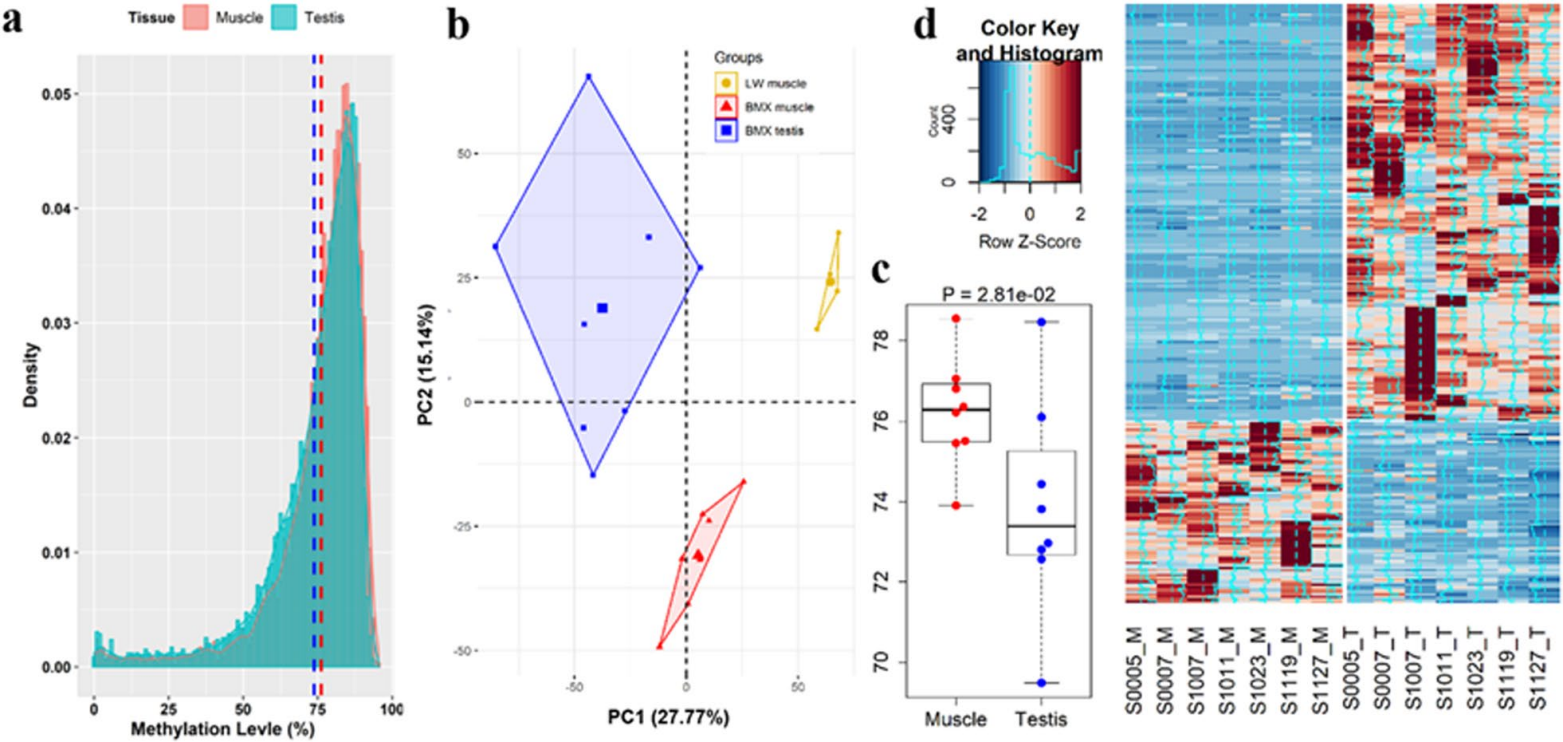
Methylation levels of proximal PRE1 correlate with the expression levels of the neighboring genes. **a** Distribution of methylation levels of proximal PRE1 copies. The dotted lines represent the overall average in muscle and testes. **b** PCA based on the methylation levels of proximal PRE1 in muscle and testes. **c** Global methylation level of proximal PRE1 of individuals. **d** Differentially-expressed genes between muscle and testes related to the mean methylation levels of proximal PRE1

To investigate the potential functions of PRE1, we scanned the average methylation level of CpG for 6124 PRE1 that are proximal to genes. Most of the proximal PRE1 maintained their hypermethylation in the two tissues examined, i.e. muscle and testis (Fig. 5a), and PCA showed that the methylation patterns of these PRE1 differed between the two tissues (Fig. 5b). The average methylation level was lower in the testis than in the muscle (deviation from mean = 2.40, T test, *P* < 0.05) and interindividual differences were larger in the testis than in the muscle (CV 3.59 vs. 1.78%, Fig. 5c). Approximately 1128 (18.4%) PRE1 that were located proximal to 1040 genes showed significant differences in methylation level between the two tissues (Wilcoxon signed rank test, *P* < 0.01), and among these genes, 247 also showed significant differential expression between these two tissues (Wilcoxon signed rank test, *P* < 0.01, Fig. 5d). Correlation analysis showed that the methylation level of 103 and 261 PRE1 were, respectively, negatively and positively correlated with gene expression (Spearman correlation, *P* < 0.01).

**Fig. 6:**
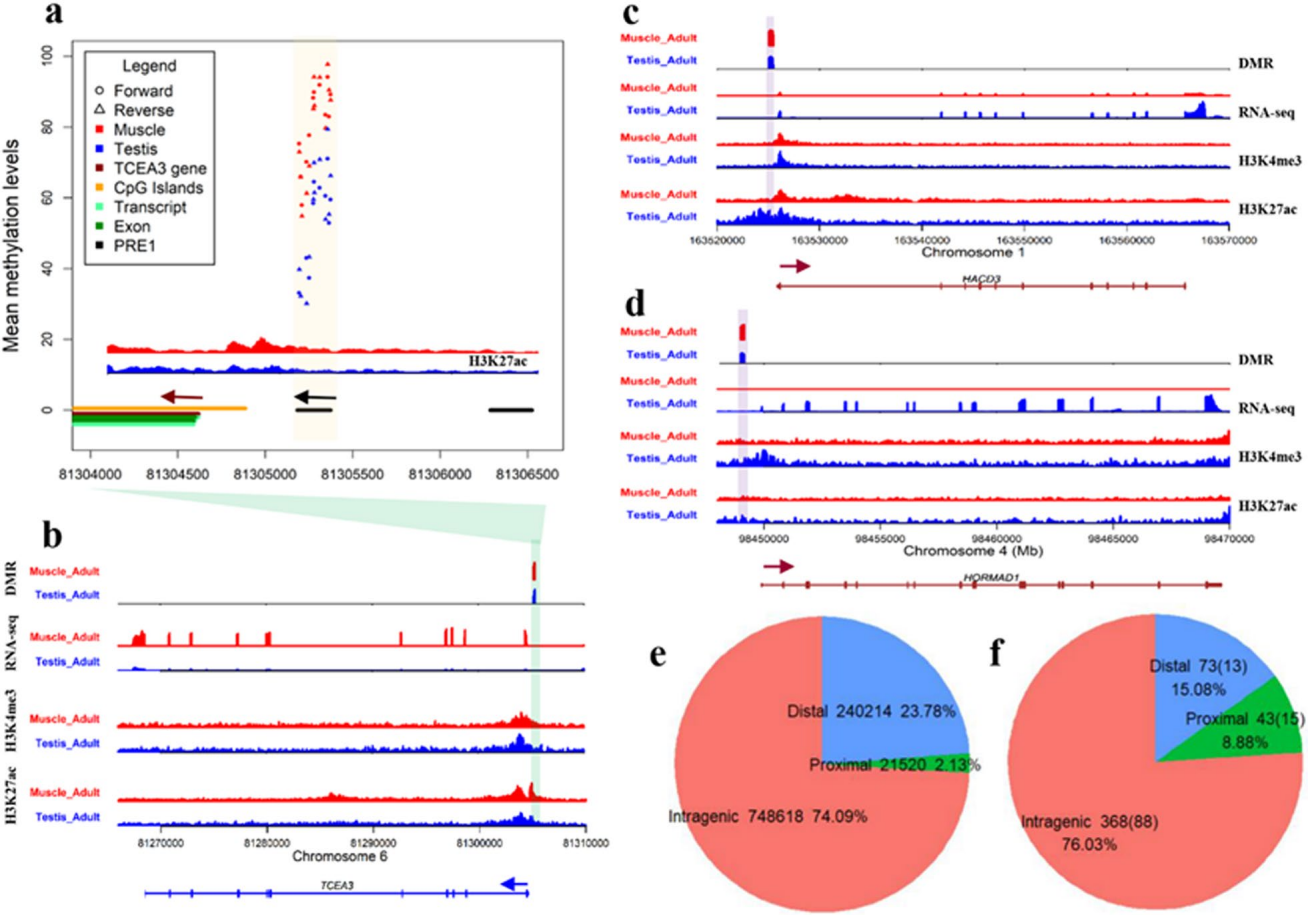
Proximal PRE1 as a *cis*-regulatory element. **a** Differentially-methylated PRE1 and H3K27ac signals in the promoter of the *TCEA3* gene. **b**–**d** Epigenetic and expressed signals in the *TCEA3*, *HACD3* and *HORMAD1* genes. **e**, **f** Number of PRE1 and differentially methylated PRE1 copies in intragenic, proximal, and distal regions. Proximal PRE1 and distal PRE1 were defined as those that are 1 to 2 kb and 2 to 100 kb upstream of their flanking genes, respectively

To demonstrate the effect of methylated PRE1 on tissue-specific regulation, we focused on the promoter region of *TCEA3* that harbors a significantly differentially methylated PRE1 between muscle and testis (Fig. 6a) and which we had previously shown to have a methylation level that was significantly positively correlated with gene expression [21]. H3K27ac signals (Fig. 6b) showed that the differentially methylated PRE1 is located on the distal side of the enhancer of the *TCEA3* gene [41]. To exclude the possibility that the H3k27ac active signal of *TCEA3* is due to interference with the transcriptional activity of PRE1 itself, we examined the RNA-seq, small RNA-seq and Iso-seq data of adult testis and muscle tissues to check the transcriptional activity of PRE1 and found that PRE1 was transcriptionally inactivated (in-house data) and no H3K4me3 peak was detected in the PRE1 regions. These results suggest that tissue-specific methylation of the *TCEA3* proximal PRE1 was conducive to enhancer-promoter activity. In addition, we observed demethylation of PRE1 in the proximal upstream regions of the *HACD3* (chr1:163525170-163525471) and *HORMAD1* (chr4:98448876-98449179) genes, which suggests a tissue-specific enhancer and promoter function [Fig. 6c, d and (see Additional file 1: Fig. S4)]. These results imply that methylation of PRE1 is related to multiple tissue-specific *cis*-regulatory functions.

Our previous studies showed that a PRE1 copy inserted into the *VRTN* gene (g.20311_20312ins291) can increase its expression nine-fold in pig embryos and can also increase the number of thoracic vertebrae [9, 42, 43]. Moreover, PRE1 activates the expression of reporter genes, both in vitro and in vivo [9].

According to a previous analysis of an intragenic PRE1 (*VRTN g.20311_20312ins291*) that promotes host gene transcription [9], most enhancers are enriched in regions that are 2 to 100 kb away from the nearest gene [44–46]. We compared the differentially-methylated PRE1 in the intragenic, proximal, and distal regions. The numbers of PRE1 in these three regions are shown in Fig. 6e. We captured the differentially-methylated PRE1 between muscle and testis by setting stricter criteria, including at least 10 filtered CpG in each PRE1 copy, a Wilcoxon signed rank test *P* < 0.01, and an absolute difference between the means of the two tissues greater than 0.3. The numbers of differentially-methylated PRE1 and differentially-expressed genes are shown in Fig. 6f. The results showed that, compared to other PRE1, proximal PRE1 are more likely to show differential methylation between muscle and testis (Fisher’s exact test, *P* < 0.01).

## Discussion

In this study, we modelled the evolution of the PRE1 sequence from the ancestral CHRS-S1 family to its current form through three successive partial duplications. Specifically, PRE1 RNA splicing models have been proposed to explain partial duplications, deletions, and degenerate direct repeats in PRE1. Epigenetic evidence shows that PRE1 may act as methylation-associated functional elements.

We propose to divide the PRE1 lineages into four subfamilies (PRE1A, PRE1B, PRE1C, and PRE1D) based on evolutionary history, which includes three successive partial duplications and degenerations of direct repeats. The partial duplication of the CHRS-1 head region leads to the ancestral PRE1 subfamily (PRE1A or PRE1l). A short partial duplication in the PRE1A body region forms the ancestral PRE1B (including PRE1i, PRE1j, and PRE1k). Similarly, a long partial duplication in the PRE1B body region produces the ancestral PRE1C (including PRE1a, PRE1b, PRE1c, PRE1d, PRE1e, PRE1f, PRE1h). PRE1D (including PRE1d2 and PRE1f2) is produced by degeneration of a direct repeat from PRE1C. Although we have redrawn the general structure of PRE1, it must be noted that the PRE1 family has accumulated a large number of different indels (Fig. 1a). A previous study reported that the PRE1a lineage contains elements that are similar, indicating very recent and possibly current activity [16]. Following the splicing model, the activity of PRE1a should contribute to the continuous formation of new PRE1 copies, with possible new duplication/deletion events.

The retrotransposition of PRE1 increases the length of genes and causes alternative splicing. We analyzed 21,420 single-copy genes that were longer than 1000 bp and found that the number of PRE1 was strongly correlated with gene length (Spearman’s *r* = 0.912, *P* < 2.2 × 10^–16^). The PRE1 copy number was moderately correlated with the number of annotated gene transcripts (Spearman’s *r* ≈ 0.5, *P* < 2.2 × 10^–16^). A large number of splicing signals was detected in PRE1 (Fig. 3e). These results showed that intragenic PRE1 may mediate alternative splicing and exonization of protein-coding genes. Strong splicing signals (GU-AG) in PRE1 provided resources for partial duplications over a long evolutionary history and is probably the phenomenon that drives complex repetitive structures in CHRS families [17]. Partial duplications and degenerations are important mechanisms for increasing the diversity and complexity of the PRE1 sequences.

SINEs are considered to be *cis*-regulatory elements [10, 47]. We used multidimensional epigenomic data to identify tissue-specific *cis*-regulatory PRE1 elements through methylation analysis of CpG in PRE1. As proof of principle, we show that three PRE1 close to the *TCEA3*, *HACD3,* and *HORMAD1* genes act as promoters or enhancers to activate or enhance gene expression by methylation. Differentially-methylated PRE1 in the muscle and testis were positively or negatively correlated with the expression of neighboring genes. This illustrates that methylated proximal PRE1 have dual functions (inhibition and activation) in the regulation of gene expression. ChIP-seq data show that these functional PRE1 elements are located in the distal flanks of enhancers, which is consistent with the previous view that enhancers that are located on either side of SINEs could provide platforms for chromatin rearrangement via the physical or biochemical properties of their sequences [41]. The functions of PRE1 as regulatory elements can be spatiotemporally specific, and DNA methylation plays an important epigenetic function to adapt the binding to specifically expressed *trans*-acting factors and achieve specific regulating effects. CpG methylation can influence the binding affinity of most transcription factors to DNA [48]. Some of these transcription factors have two functions depending on the tissue and act as silencers and enhancers in different cellular contexts [49]. PRE1 subfamilies are enriched for specific TF motifs, and not every PRE1 can be effectively bound by those TF because of epigenetic modification, co-selection, and mutation [10]. In conclusion, the unique origin and evolution of PRE1 provide a structural basis for diverse regulatory functions. More research is needed to identify and differentiate context-, motif- or lineage-specific functions, and multifunctional PRE1 copies.

Through this study, we summarized several features of PRE1 as diverse *cis*-regulatory elements. First, most PRE1 are inactive and have lost their transposition ability during their evolution [14, 16]. Most extant PRE1 insertions are neutral to the host gene and have become fixed through genetic drift [10]. The three partial duplications of the tRNA promotor for the PRE1 body amplified the affinity of promoter transcription factors (Fig. 2a). Thus, it naturally follows that PRE1 near the host gene may interfere with its expression. Second, some PRE1 have accumulated many CG dinucleotides (Fig. 1c, d), which can act as CpG islands to increase or decrease gene expression by methylation [48, 50, 51]. Third, the PRE1 family has evolved into abundant lineages through single nucleotide variation, insertion, deletion, and partial duplication (Figs. 1a and 2a). These different lineages might have acquired specific TF-binding motifs [10]. Finally, the million or more PRE1 in the genome can physically interact with other regulatory elements and promoters and form stable 3D structures [47].

In addition, intragenic PRE1 are involved in posttranscriptional regulation processes. First, most splicing sites are enriched in PRE1 (Fig. 3e), and PRE1 in introns of transcribed genes mediate exonization and alternative splicing. Second, we predicted that PRE1 RNA has a complex spatial structure (Fig. 2b) and that many PRE1 are proximal to the 3′ and 5′ UTR of genes (Fig. 3d). PRE1 in the 5′ UTR of mRNA can suppress translation by forming secondary structures [52], and PRE1 in the 3′ UTR can increase the stability of the mRNA. Third, we found that two miRNA (ssc-miR-4331-1 and ssc-miRNA-4331-2) are derived from PRE1 and may regulate homologous PRE1-derived target sites in the 3′ UTR of the mRNA. Finally, PRE1 has other functions similar to those of the human SINEs (Alu) [47].

## Conclusions

In this study, we focused on the origin, evolution and functions of porcine PRE1. PRE1 have originated from CHRS-1 through three main partial duplications. The PRE1 family has accumulated many structural variations including duplications, deletions, insertions, and degenerate direct repeats over a long history of propagation. In particular, the late evolution of PRE1 was accompanied by the degeneration of direct repeats. We also observed many splicing sites that are located within intragenic PRE1. These splicing sites in single PRE1 and in nested and tandem PRE1 have contributed to the evolution of PRE1. We inferred a PRE1 RNA splicing model, which may explain the formation of partial duplications and degenerate direct repeats. In terms of *cis*-regulatory functions, we found that PRE1 sequences act as methylation-associated tissue-specific enhancers or promoters.

## Supplementary Information

**Additional file 1: Figure S1. **Nested and tandem PRE1. **a** Mechanisms of the formation of nested PRE1 and tandem PRE1. **b** A PRE1 inserted into another PRE1 tail. The target motif is located in the PRE1 tail (chr7:29710723:29711249). The nested PRE1 are actually two new tandem PRE1 with changed tile lengths. The tail of the basal PRE1 at the 5′ end is shortened, but the tail of the embedded PRE1 at the 3′ end is extended. Target site duplications (TSD) are shown in red font. Embedded PRE1 are shown in blue font, and basal PRE1 are shown in green font. **c** Repeated TSD produce three tandem PRE1 (chr12:33294823-33295640); **d** A PRE1 inserted into another PRE1 body. The target motif is located in the PRE1 body, which leads to a nested PRE1 (chr14:22207023-22207668); and **e** A tandem PRE1 inserted into the genome (chr2:80470307-80470742). **Figure S2.** Internal repeat segments in PRE1. **a** The consensus sequence of quasi-oligomeric PRE1 including three direct repeats. The black arrows indicate the three direct repeats; **b** Several rare PRE1 with other direct repeats in the PRE1 body. Four repeat segments of PRE1 (chr15:28050427-28050730, chr6:166491294-166491728, chr7:65896533-65896843; and chr15:77600095-77600373) have been marked in the consensus sequence. The arrows show the duplicated segments in different PRE1; and **c** The degenerate 46-bp repeat sequence in PRE1. The blue dotted arrows show the degenerate 46-bp repeat sequence, and the green arrows show the direct repeats. **Figure S3.** Three alternative alignment methods for PRE1d2. The PRE1d2 shows a degenerate 46-bp segment following the splicing model, and PRE1d2(2) and PRE1d2(3) show hypothetically missed whole R or F direct repeats, respectively. **Figure S4.** Differentially-methylated PRE1 in the promoter regions of the *HACD3* and *HORMAD1* genes.

## Data Availability

The methylation and RNA-seq data of the 16 samples (8 testes and 8 muscles) have been deposited in the China National center for Bioinformation with project ID PRJCA003221 (https://bigd.big.ac.cn/bioproject/browse/PRJCA003221).
