# Supplementary material for: Origin, evolution, and tissue-specific functions of the porcine repetitive element 1
Source: Genet Sel Evol. 2022 Jul 27;54:54. doi: 10.1186/s12711-022-00745-3 (PMC9327148; doi:10.1186/s12711-022-00745-3)

**Additional file 1 Figure S1**


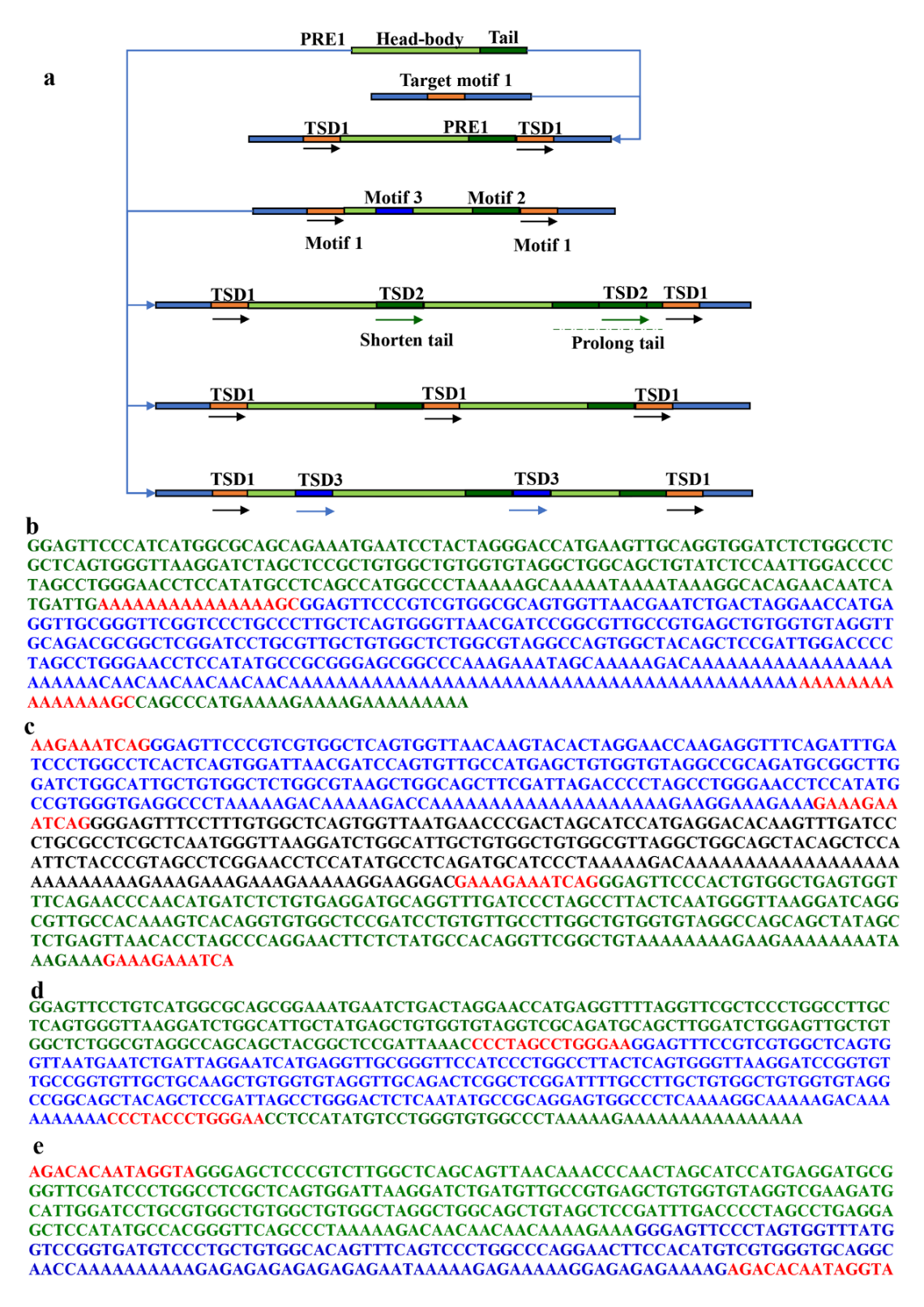


**Additional file 1 Figure S2**


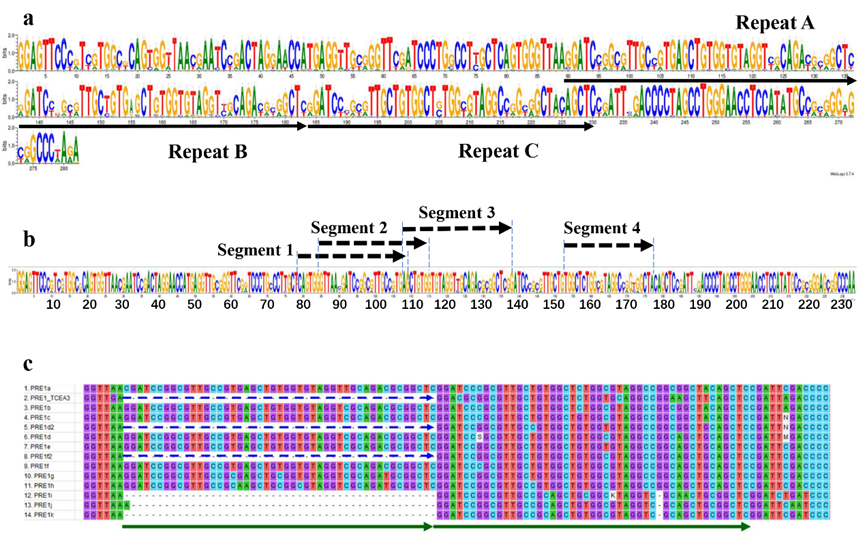


**Additional file 1 Figure S3**


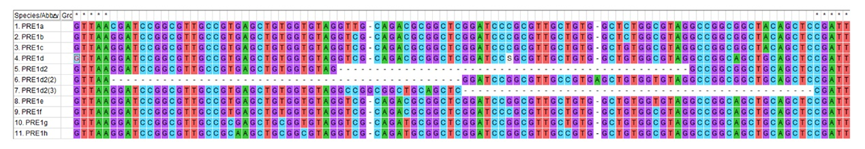


**Additional file 1 Figure S4**


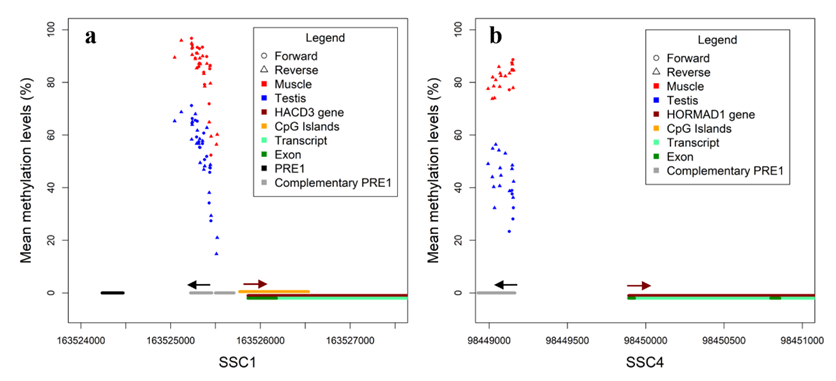

Supplement: Supplementary file 1 — Additional file 1: Figure S1. Nested and tandem PRE1. a Mechanisms of the formation of nested PRE1 and tandem PRE1. b A PRE1 inserted into another PRE1 tail. The target motif is located in the PRE1 tail (chr7:29710723:29711249). The nested PRE1 are actually two new tandem PRE1 with changed tile lengths. The tail of the basal PRE1 at the 5′ end is shortened, but the tail of the embedded PRE1 at the 3′ end is extended. Target site duplications (TSD) are shown in red font. Embedded PRE1 are shown in blue font, and basal PRE1 are shown in green font. c Repeated TSD produce three tandem PRE1 (chr12:33294823-33295640); d A PRE1 inserted into another PRE1 body. The target motif is located in the PRE1 body, which leads to a nested PRE1 (chr14:22207023-22207668); and e A tandem PRE1 inserted into the genome (chr2:80470307-80470742). Figure S2. Internal repeat segments in PRE1. a The consensus sequence of quasi-oligomeric PRE1 including three direct repeats. The black arrows indicate the three direct repeats; b Several rare PRE1 with other direct repeats in the PRE1 body. Four repeat segments of PRE1 (chr15:28050427-28050730, chr6:166491294-166491728, chr7:65896533-65896843; and chr15:77600095-77600373) have been marked in the consensus sequence. The arrows show the duplicated segments in different PRE1; and c The degenerate 46-bp repeat sequence in PRE1. The blue dotted arrows show the degenerate 46-bp repeat sequence, and the green arrows show the direct repeats. Figure S3. Three alternative alignment methods for PRE1d2. The PRE1d2 shows a degenerate 46-bp segment following the splicing model, and PRE1d2(2) and PRE1d2(3) show hypothetically missed whole R or F direct repeats, respectively. Figure S4. Differentially-methylated PRE1 in the promoter regions of the HACD3 and HORMAD1 genes. [file 12711_2022_745_MOESM1_ESM.docx]
